# Peer Review of “Prevalence and Determinants of Academic Bullying Among Junior Doctors in Sierra Leone: Cross-Sectional Study”

**DOI:** 10.2196/75135

**Published:** 2025-05-22

**Authors:** Jenny Wilkinson

**Affiliations:** 1 Metavision Institute, Brisbane City, Australia

**Keywords:** academic bullying, junior doctors, Sierra Leone, mental health, professional development

*This is the peer-review report for “Prevalence and Determinants of Academic Bullying Among Junior Doctors in Sierra Leone: Cross-Sectional Study.”*

## Round 2 Review

### General Comments

This study [1] presents a survey of junior doctors in Sierra Leone hospitals and their experience of bullying and found high levels of bullying among the participants. Below are comments and suggestions for clarifying and strengthening the work.

### Specific Comments

#### Major Comments

1. The author’s definition of bullying and whether it was provided to participants is somewhat unclear. In the abstract, bullying is described as involving repeated behaviors, which aligns with the typical definition of bullying as an ongoing or repeated action. However, in the Methods section, participants were asked to respond based on any instance of various behaviors. While a single act of intimidation, for example, constitutes inappropriate behavior that should be addressed, it may not meet the standard definition of bullying. It is essential to clarify this distinction and ensure that participants also recognized the difference so that general poor behavior is not conflated with bullying.
2. Was sampling randomly, equally, or proportionally distributed across the four sites, and were there any analyses done based on site?
3. How was random sampling achieved?
4. Please comment on the reliability and validity of the instrument used to collect data. What literature was used to inform the development of the questions? Please include this information in the manuscript.
5. At the start of paragraph 3 of the Introduction, the authors refer to “other contexts”; it is unclear what contexts are being referred to in this and the preceding paragraph.
6. The Introduction and Discussion would be strengthened by more specific references to literature findings. I found the text in both a little superficial.
7. It is unclear whether the participants were reporting behaviors they personally experienced (ie, they were bullied) against behaviors they observed (ie, others being bullied).
8. Please provide clarification as to who is a “junior doctor.” This journal has an international readership, and this term can be used differently in different countries, with “junior doctors” having different lengths of service. Please ensure this is clear within the body of the manuscript.
9. The description of the multiple regression seems a little excessive given the lack of statistical significance. This could be made more concise and simply refer readers to Table 3. Similarly, the authors should be cautious not to overemphasize these findings.
10. The list of references needs to be reviewed to ensure that all items have full bibliographic details.

## Round 3 Review

The authors have addressed the review comment in their response, and this has been somewhat translated to the manuscript itself, noting that the lack of track changes, list of specific changes, or other highlights of manuscript revisions makes it difficult to see what changes were made. For example, while the comments regarding instrument development are addressed in the authors’ response, it is unclear whether any changes have been made to the manuscript itself.

## References

[1] Jalloh F, Bah AT, Kanu A (2025). Prevalence and determinants of academic bullying among junior doctors in Sierra Leone: cross-sectional study. JMIRx Med.

